# Identification of suitable controls for miRNA quantification in T-cells and whole blood cells in sepsis

**DOI:** 10.1038/s41598-019-51782-w

**Published:** 2019-10-31

**Authors:** Simon Hirschberger, Max Hübner, Gabriele Strauß, David Effinger, Michael Bauer, Sebastian Weis, Evangelos J. Giamarellos-Bourboulis, Simone Kreth

**Affiliations:** 1 Department of Anaesthesiology and Intensive Care Medicine, University Hospital, Ludwig Maximilian University (LMU), Munich, Germany; 2 Walter-Brendel-Center of Experimental Medicine, Ludwig Maximilian University (LMU), Munich, Germany; 3 Department of Anaesthesiology and Intensive Care Medicine, Friedrich-Schiller University, Jena, Germany; 4 Center for Sepsis Control and Care, Jena University Hospital, Jena, Germany; 5 Institute for Infectious Disease and Infection Control, Jena University Hospital, Jena, Germany; 6 4th Department of Internal Medicine, ATTIKON University Hospital, National and Kapodistrian University of Athens, Athens, Greece

**Keywords:** Translational research, Molecular medicine, miRNAs, Experimental models of disease

## Abstract

Complex immune dysregulation is a hallmark of sepsis. The occurring phases of immunosuppression and hyperinflammation require rapid detection and close monitoring. Reliable tools to monitor patient’s immune status are yet missing. Currently, microRNAs are being discussed as promising new biomarkers in sepsis. However, no suitable internal control for normalization of miRNA expression by qPCR has been validated so far, thus hampering their potential benefit. We here present the first evaluation of endogenous controls for miRNA analysis in human sepsis. Novel candidate reference miRNAs were identified via miRNA microArray. TaqMan qPCR assays were performed to evaluate these microRNAs in T-cells and whole blood cells of sepsis patients and healthy controls in two independent cohorts. In T-cells, U48 and miR-320 proved suitable as endogenous controls, while in whole blood cells, U44 and miR-942 provided best stability values for normalization of miRNA quantification. Commonly used snRNA U6 exhibited worst stability in all sample groups. The identified internal controls have been prospectively validated in independent cohorts. The critical importance of housekeeping gene selection is emphasized by exemplary quantification of imuno-miR-150 in sepsis patients. Use of appropriate internal controls could facilitate research on miRNA-based biomarker-use and might even improve treatment strategies in the future.

## Introduction

Sepsis is a dysregulated immune response to host infection causing severe organ dysfunction^1^. To date, it remains a life-threatening condition with high mortality rates^2,3^. Although originally considered to present with a biphasic immune response, it is nowadays accepted that sepsis is characterized by a complex immune dysregulation of both innate and adaptive immunity, with transitory immunosuppression and hyperinflammation emerging alternately or simultaneously^4,5^. Although various biomarkers have been proposed^6^, effective tools for monitoring patients’ immune status are still missing, thus hampering targeted therapy of sepsis. In this situation, microRNAs have been suggested as biomarkers in sepsis^7^.

MicroRNAs are a class of small non-coding RNA molecules that exert pivotal biological functions through post-transcriptional regulation of cellular gene expression^8^. By base-pairing to specific recognition sites within the 3′ untranslated region (UTR) of their respective target mRNAs they either repress translation or enable mRNA degradation^9,10^. MicroRNAs are remarkably stable and easily measurable^7,11^. They are tissue- and cell-type-dependently expressed and specific changes in miRNA expression due to cellular damage create disease-specific miRNA signatures^4,12,13^. These features render microRNAs ideal biomarkers^14^.

Innate and adaptive immune cells are regulated by specific miRNAs -so called immuno-miRs- that create a versatile balance of pro- and anti-inflammatory signalling circuitries^15^. Consequently, immuno-miRs have gained attraction in sepsis research and numerous studies have been conducted to identify immuno-miRs that could serve as biomarkers to determine the inflammatory state of the individual sepsis patient^7,16^. Results, however, were heterogeneous thus currently impeding the use of miRNA as biomarkers. This lack of consensus might -at least partially- be due to the fact that valid reference genes for quantification of miRNAs in sepsis have not been established, yet^17–23^. Moreover, in many cases either plasma or serum samples were used, which bears the risk of detecting contaminating miRNAs induced or released by co-morbidities or environmental influences.

We here present the first validation of internal controls for qPCR-based miRNA quantification in immune cells in sepsis. Importantly, to address the divergent miRNAomes of immune cell types, we have investigated reference miRNAs for two relevant set-ups: T-cells and whole blood cells. We analyzed native and stimulated T-cells from healthy volunteers and T cells isolated from septic patients and newly identified two reliable internal reference miRNAs. To expand these findings to PAXgene bed-side application, whole blood cells from a second, independent cohort of sepsis patients and healthy controls were isolated, and two valid reference miRNAs were identified also for this scenario.

## Results

### Selection of candidate miRNA reference genes

An optimal housekeeping gene should fulfil several essential demands: (1) it is highly expressed in the target cell, (2) it shows no differential regulation thus exhibiting high expression stability over time, (3) it is unaffected by any experimental condition and (4) it is easily detectable by use of available assays^24,25^. Taking into account the highly cell- and tissue-specific expression of miRNAs, it is intuitively clear that universal reference genes do not exist. Instead, optimal internal controls according to these requirements have to be determined for each utilized type of cell or tissue and every experimental approach^20,26,27^.

To identify potential miRNAs/snRNAs/snoRNAs that could serve as endogenous controls for quantification of miRNA expression in T-cells and whole blood cells in sepsis, we first evaluated data from a miRNA microArray performed with RNA from T-cells of septic patients and unstimulated/stimulated T-cells of healthy controls. Expression levels of candidate miRNAs are shown in Fig. S1. Requirements for suitable miRNAs were: (i) Expression detectable in at least 19/21 samples, (ii) not differentially regulated in sepsis (p-value > 0.05) and (iii) TaqMan miRNA assay commercially available^28^. With respect to these criteria, three microRNAs fulfilled all preconditions and thus were included into subsequent analyses: miR-30c-1*, miR-320a and miR-942. In addition, the commonly used albeit not properly evaluated normalizers U6, U44, U47 and U48 were analysed (Table 1). These seven candidate reference genes where then further assessed in two independent cohorts: (1) T-cells of septic patients and age-adjusted healthy controls (native and activated via anti-CD3/CD28 microBeads) and (2) whole blood cells of septic patients and age-adjusted healthy controls. Patient characteristics are depicted in Supplementary Tables 2–5.

**Table 1:** TaqMan miRNA assays.

Target	Assay-Nr.	Accession number
RNU6B	001093	NR_002752
RNU44	001094	NR_002750
U47	001223	AF141346
RNU48	001006	NR_002745
hsa-miR-320a-3p	002277	MI0000542
hsa-miR-942-5p	002187	MI0005767
hsa-miR-30c-1*	002108	MI0000736

### Evaluation of candidate miRNA reference genes in T-cells

Recently, paralysis of adaptive immunity has evolved as the major cause of death in sepsis^29,30^. On the search for effective biomarkers for early detection and consecutive monitoring of immunosuppression, miRNAs have entered the field^16^. However, valid internal normalizers to enable reliable miRNA quantification have not been determined so far.

**Figure 1:**
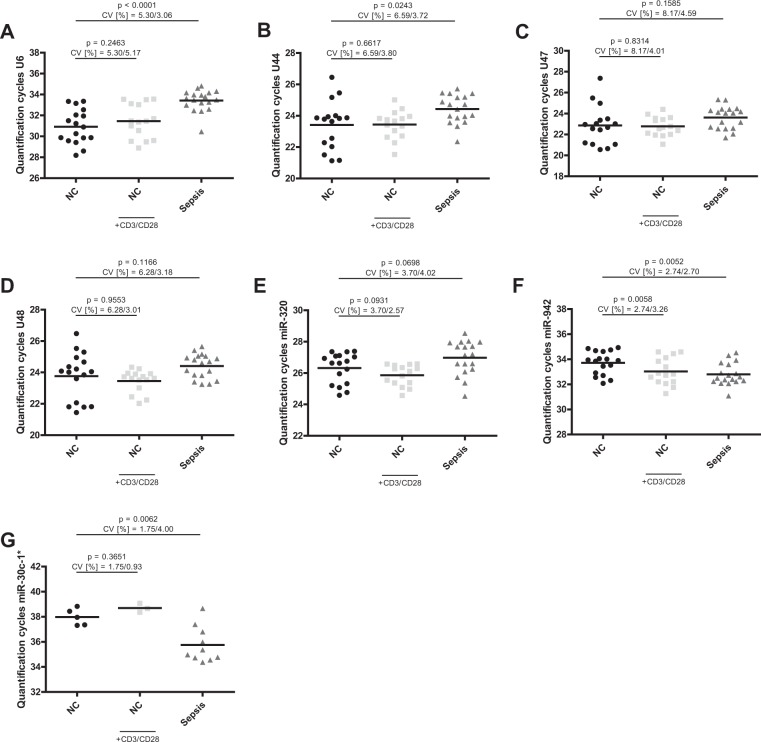
Quantification cycles of candidate internal normalizers in T-cells. Quantification cycles of (**A**) U6, (**B**) U44, (**C**) U47, (**D**) U48, (**E**) miR-320a, (**F**) miR-942 and (**G**) miR-30c-1* were assessed in T-cells of sepsis patients and native and CD3/CD28-activated T-cells from age-adjusted healthy controls. Data are shown as mean with range. n = 18/17 (Sepsis/NC), miR-30c-1* n = 10/5 (Sepsis/NC).

To address this important issue, we evaluated expression levels of the seven previously defined candidate miRNAs in T-cells from septic patients (n = 18) and in both native and stimulated T-cells from healthy controls (n = 17) (Fig. 1). Raw quantification cycles (Cq) ranged from 23 to 34 (Table 2 shows raw Cq values and standard deviation (SD) of each subset, amplification efficiencies are depicted in Supplementary Table 1). Only MicroRNA-30c-1* exhibited higher quantification cycles (in the range of 36–38), indicating only unspecific amplification, and thus was excluded from further analyses.

**Table 2:** Mean raw Cq and SD for candidate miRNA internal normalizers in T-cells.

miRNA	Healthy controls		Healthy controls anti-CD3/CD28		Sepsis patients	
	Mean Cq	SD	Mean Cq	SD	Mean Cq	SD
U6	30.92	1.639	31.45	1.626	33.42	1.023
U44	23.42	1.543	23.44	0.8913	24.43	0.9093
U47	22.87	1.867	22.78	0.9135	23.61	1.083
U48	23.77	1.493	23.45	0.7055	24.41	0.7760
miR-30c-1*	37.98	0.6651	38.70	0.3607	35.76	1.429
miR-320a	26.32	0.9726	25.86	0.6649	26.97	1.083
miR-942	33.71	0.9237	33.03	1.076	32.80	0.8868

Mean Cq = mean quantification cycle; SD = standard deviation of Cq values.

#### Reference genes for miRNA quantification in native vs. stimulated primary human T-cells

As *ex vivo* stimulation of primary T-cells is a commonly used and generally accepted method to mimic global activation of T-cells in response to infection, we first investigated expression stability of the remaining six candidate miRNAs in unstimulated and in anti-CD3/CD28 stimulated primary T-cells of healthy donors. To this end, we used the open-source algorithms NormFinder, BestKeeper and geNorm. Standard deviations and gene stability values of these analyses are shown in Table 3. Fold change of miRNA expression is depicted in Fig. S4 and Supplementary Table 6. Rankings of all applied algorithms are depicted in Table 4.

**Table 3:** Results of BestKeeper, NormFinder and geNorm analysis for candidate microRNA internal controls in naive and stimulated primary T-cells.

miRNA	U6	U44	U47	U48	miR-320	miR-942
geo Mean [Cq]*	31.19	23.40	22.78	23.50	26.07	33.33
ar Mean [Cq]*	31.23	23.43	22.82	23.53	26.08	33.35
min Cq [Cq]*	28.18	21.13	20.55	21.45	24.57	31.27
max Cq [Cq]*	33.57	26.45	27.37	25.53	27.39	34.93
SD [±Cq]*	1.36	0.96	1.06	0.85	0.73	0.89
CV [% Cq]*	4.34	4.12	4.65	3.59	2.82	2.66
stability value^#^	0.343	0.125	0.153	0.095	0.125	0.190
best combination^#^	U44 and U48 | combined stability value: 0.084					
*M* stability value	1.910	0.951	1.119	0.957	0.949	1.039

geo Mean = geometric mean of Cq values; ar Mean = arithmetic mean of Cq values; min Cq = minimal Cq; max Cq = maximal Cq; SD = standard deviation of Cq values; CV = coefficient variation. *BestKeeper results. ^#^NormFinder results. *M* stability value = geNorm results.

**Table 4:** Stability ranking of candidate reference genes in naive and stimulated primary T-cells.

Rank	NormFinder	BestKeeper	geNorm
1	U48	miR-320	miR-320
2	miR-320/ U44	U48	U44
3		miR-942	U48
4	U47	U44	miR-942
5	miR-942	U47	U47
6	U6	U6	U6

**Figure 2:**
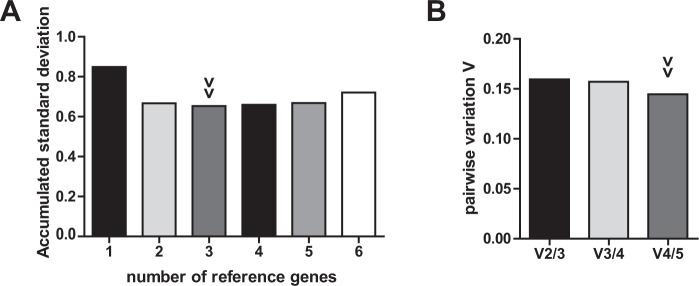
Optimal number of reference genes for normalization of stimulated primary T-cells. (**A**) Accumulated standard deviation and (**B**) pairwise variation calculations (V_n_/V_n+1_) of native and CD3/CD28-activated primary T-cells of healthy donors according to geNorm and GenEx software applications. Arrowheads indicate optimal number of reference genes.

MicroRNA-320 exhibited best performance according to BestKeeper and geNorm analysis. NormFinder identified U48 as most stable gene and U48/U44 as best combination of genes. However, both miR-320 and U48 proved as reliable with only marginal differences and high performance throughout all analyses. Notably, commonly used U6 was ranked worst by all applied algorithms. The combined use of three or even two reference genes resulted in only marginally higher yet similar V_n_/V_n+1_ ratios. In accordance to these findings, accumulated standard deviations are comparably low for two to four reference genes (Fig. 2). Taken together, miR-320 or the combination of miR-320 and U48 are suitable as internal controls of miRNA expression analysis in naive and stimulated primary human T-cells.

#### Reference genes for miRNA quantification in septic T-cells vs. healthy controls

**Figure 3:**
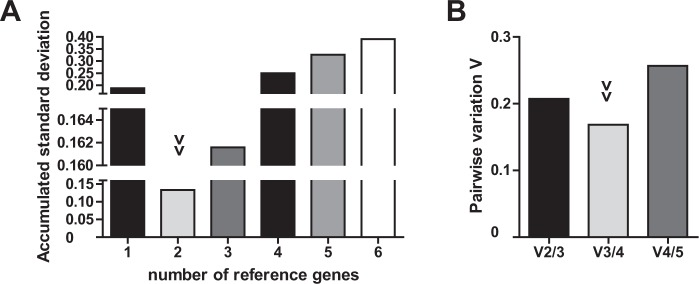
Optimal number of reference genes for normalization of septic T-cells. (**A**) Accumulated standard deviation and (**B**) pairwise variation calculations (V_n_/V_n+1_) of septic T-cells and age-adjusted healthy controls according to geNorm and GenEx software applications. Arrowheads indicate optimal number of reference genes.

In clinical settings, evaluation of T-cell miRNAs in sepsis is based on comparison of sepsis samples to healthy controls. We thus determined Cq values of all candidate miRNA internal normalizers in T-cells of sepsis patients and in healthy controls and analysed their expression stability. Results are shown in Table 5, respective rankings are listed in Table 6. While U48 exhibited best stability values proposed by NormFinder and geNorm, miR-942 showed superior performance according to BestKeeper analysis. NormFinder combination analysis revealed miR-320 and U48 as best performing genes. Overall, values of U48 and miR-320 were in close proximity throughout all calculations. Again, U6 was consistently ranked worst. The combined use of two internal controls exhibited the best stability values (Fig. 3). In summary, applying U48 or the combination of miR-320 and U48 provides best internal normalization for miRNA quantification when comparing primary T-cells of sepsis patients to those of healthy controls.

**Table 5:** Results of BestKeeper, NormFinder and geNorm analysis for candidate microRNA internal controls in healthy and septic primary T-cells.

miRNA	U6	U44	U47	U48	miR-320	miR-942
geo Mean [Cq]*	32.24	26.63	23.21	24.00	26.63	33.18
ar Mean [Cq]*	32.39	23.95	23.26	24.03	26.65	33.19
min Cq [Cq]*	28.18	21.13	20.55	21.45	24.53	31.08
max Cq [Cq]*	34.81	26.45	27.37	25.65	28.54	34.93
SD [±Cq]*	1.50	1.00	1.20	0.88	0.88	0.86
CV [% Cq]*	4.65	4.16	5.15	3.68	3.28	2.59
stability value^#^	0.743	0.176	0.226	0.079	0.099	0.697
best combination^#^	miR-320 and U48|combined stability value: 0.076					
*M* stability value	1.814	0.994	1.178	0.973	1.016	1.525

geo Mean = geometric mean of Cq values; ar Mean = arithmetic mean of Cq values; min Cq = minimal Cq; max Cq = maximal Cq; SD = standard deviation of Cq values; CV = coefficient variation. *BestKeeper results. ^#^NormFinder results. *M* stability value = geNorm results.

**Table 6:** Stability ranking of candidate reference genes in healthy and septic primary T-cells.

Rank	NormFinder	BestKeeper	geNorm
1	U48	miR-942	U48
2	miR-320	miR-320	U44
3	U44	U48	miR-320
4	U47	U44	U47
5	miR-942	U47	miR-942
6	U6	U6	U6

**Figure 4:**
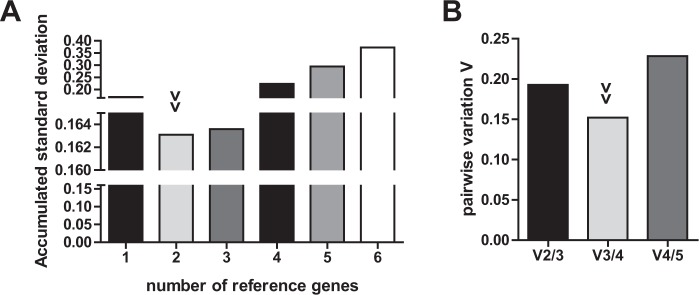
Optimal number of reference genes for normalization of T-cells. (**A**) Accumulated standard deviation and (**B**) pairwise variation calculations (V_n_/V_n+1_) of septic T-cells and native/CD3CD28-activated age-adjusted healthy controls according to geNorm and GenEx software applications. Arrowheads indicate optimal number of reference genes.

Since both analyses identified miR-320 and U48 as best performing internal normalizers, we next validated if they are indeed suitable “universal” reference genes for primary human T cells in the context of sepsis and thus usable in experimental as well as in clinical settings. Standard deviations and gene stability values of the respective analyses are given in Table 7, integrated rankings of all applied algorithms in Table 8. Both NormFinder and BestKeeper showed miR-320 and U48 as top performing candidate genes exhibiting comparable results. GeNorm analysis found best stability values for U44, U48 and miR-320, all exhibiting similar performance. A combination of two reference genes depicted lowest accumulated standard deviation and sufficient pairwise variation ratio (Fig. 4).

**Table 7:** Results of BestKeeper, NormFinder and geNorm analysis for candidate microRNA internal controls in primary human T-cells.

miRNA	U6	U44	U47	U48	miR-320	miR-942
geo Mean [Cq]*	31.93	23.85	23.21	23.88	26.40	33.16
ar Mean [Cq]*	31.98	23.89	23.27	23.90	26.42	33.18
min Cq [Cq]*	28.18	21.13	20.55	21.45	24.53	31.08
max Cq [Cq]*	34.81	28.34	30.94	26.47	28.54	34.93
SD [±Cq]*	1.53	0.92	1.07	0.81	0.84	0.88
CV [% Cq]*	4.78	3.85	4.64	3.38	3.17	2.65
stability value^#^	0.603	0.147	0.184	0.092	0.130	0.545
best combination^#^	U44/miR-320 | combined stability value: 0.095					
*M* stability value	1.791	0.939	1.076	0.940	0.961	1.404

geo Mean = geometric mean of Cq values; ar Mean = arithmetic mean of Cq values; min Cq = minimal Cq; max Cq = maximal Cq; SD = standard deviation of Cq values; CV = coefficient variation. *BestKeeper results. ^#^NormFinder results. *M* stability value = geNorm results. Comprehensive gene stability = RefFinder results.

**Table 8:** Integrated stability ranking of candidate reference genes in primary human T-cells.

Rank	NormFinder	BestKeeper	geNorm
1	U48	U48	U44
2	miR-320	miR-320	U48
3	U44	miR-942	miR-320
4	U47	U44	miR-942
5	miR-942	U47	U47
6	U6	U6	U6

Collectively, miR-320 and U48 proved as suitable internal controls for miRNA expression analysis in primary human T-cells in the context of sepsis.

### Evaluation of candidate miRNA internal normalizers in whole blood samples

**Figure 5:**
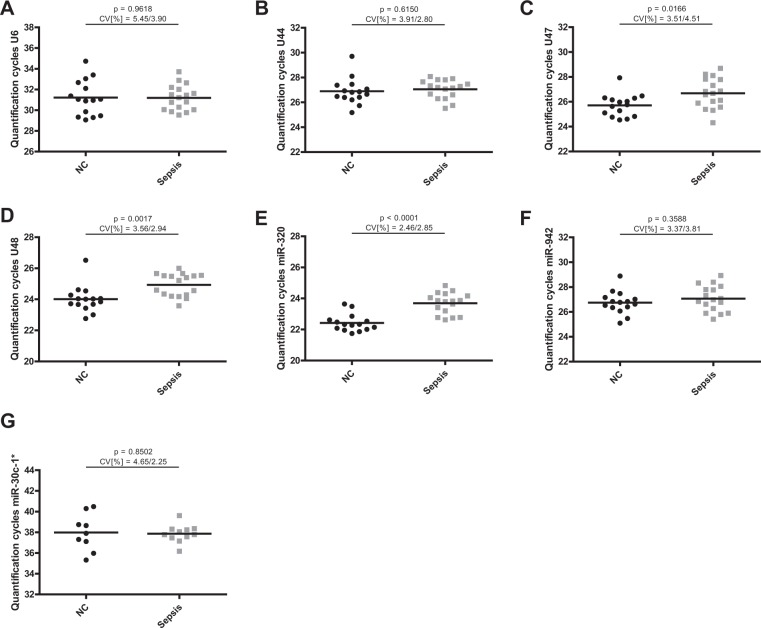
Quantification cycles of candidate internal normalizers in whole blood cells. Quantification cycles of (**A**) U6, (**B**) U44, (**C**) U47, (**D**) U48, (**E**) miR-320a, (**F**) miR-942 and (**G**) miR-30c-1* were assessed in whole blood cell RNA of sepsis patients and age-adjusted healthy controls. Data are shown as mean with range. n = 17/15 (Sepsis/NC), miR-30c-1* n = 11/9 (Sepsis/NC).

In a clinical setting, analysis of whole blood cells is a straightforward approach as the purification of single immune cell populations usually is not feasible. However, reliable references for miRNA quantification in this context are also lacking. We therefore examined expression levels of the above-mentioned candidate endogenous controls in total RNA purified from whole blood cells in an independent cohort of septic patients (n = 17) and healthy controls (n = 15, Fig. 5). Quantification cycles ranged from 23 to 32 (Table 9 shows raw Cq values and standard deviation (SD) of each subset). Similar to T-cells, miR-30c-1* was excluded from further analyses because of unspecific amplification (Cq values in the range of 37–38).

**Table 9:** Mean raw Cq and SD for candidate miRNA internal normalizers in whole blood cells.

miRNA	Healthy controls		Sepsis patients	
	Mean Cq	SD	Mean Cq	SD
U6	32.22	1.701	31.20	1.218
U44	26.89	1.050	27.05	0.7577
U47	25.71	0.9022	26.68	1.203
U48	24.01	0.8550	24.93	0.7320
miR-30c-1*	37.98	1.766	37.87	0.8536
miR-320a	22.42	0.5513	23.69	0.6741
miR-942	26.75	0.9012	27.07	1.032

Mean Cq = mean quantification cycle; SD = standard deviation of Cq values.

**Figure 6:**
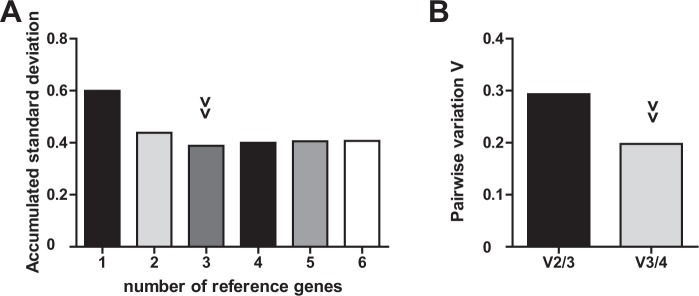
Optimal number of reference genes for normalization of whole blood cells. (**A**) Accumulated standard deviation and (**B**) pairwise variation calculations (V_n_/V_n+1_) of whole blood cells of septic patients and age-adjusted healthy controls according to geNorm and GenEx software applications. Arrowheads indicate optimal number of reference genes.

Results of expression stability displayed high consensus between all software applications: NormFinder proposes miR-942 as best internal control, whereas U44 is most suitable according to BestKeeper and geNorm. Performance of both candidate genes U44 and miR-942 is highly comparable throughout all algorithms, geNorm results are almost identical (Tables 10 and 11). Similar to T-cell analysis, U6 exhibited worst gene stability in nearly all applied software algorithms. Considering calculation of accumulated standard deviations, at least two internal controls are required (Fig. 6).

**Table 10:** Results of BestKeeper, NormFinder and geNorm analysis in whole blood cells.

	U6	U44	U47	U48	miR-320	miR-942
geo Mean [Cq]*	31.18	26.96	26.20	25.47	23.08	26.90
ar Mean [Cq]*	31.21	26.98	26.22	25.51	23.10	26.92
min Cq [Cq]*	29.07	25.18	24.31	22.77	21.74	25.10
max Cq [Cq]*	34.74	29.70	28.69	28.89	24.83	28.93
SD [±Cq]*	1.15	0.68	0.92	1.20	0.78	0.75
CV [% Cq]*	3.70	2.52	3.51	4.70	3.38	2.79
stability value^#^	0.338	0.232	0.292	0.261	0.321	0.188
best combination^#^	miR-942 and U47 | combined stability value: 0.136					
*M* stability value	1.620	1.189	1.377	1.545	1.257	1.190

geo Mean = geometric mean of Cq values; ar Mean = arithmetic mean of Cq values; min Cq = minimal Cq; max Cq = maximal Cq; SD = standard deviation of Cq values; CV = coefficient variation. *BestKeeper results. ^#^NormFinder results. M stability value = geNorm results. Comprehensive gene stability = RefFinder results.

**Table 11:** Integrated stability ranking of candidate reference genes in whole blood cells.

Rank	NormFinder	BestKeeper	geNorm
1	miR-942	U44	U44
2	U44	miR-942	miR-942
3	U48	miR-320	miR-320
4	U47	U47	U47
5	miR-320	U6	U48
6	U6	U48	U6

Taken together, our evaluation revealed miR-942 and U44 as best suitable internal controls for miRNA quantification of whole blood cell RNA in the context of sepsis.

### Validation of the identified miRNA endogenous controls

**Figure 7:**
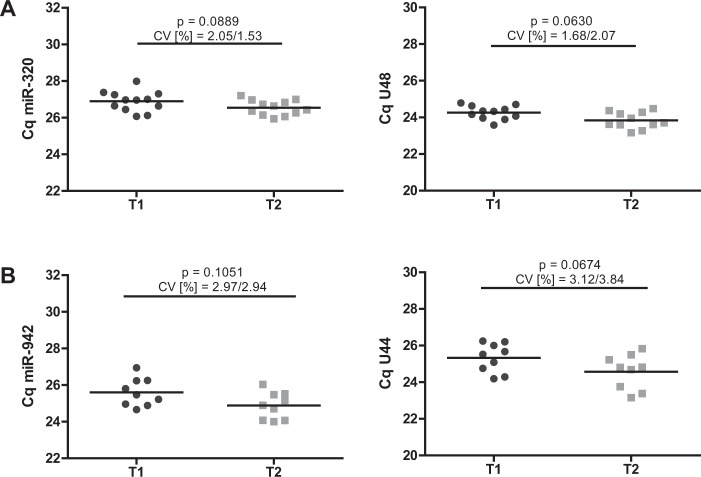
Validation of identified miRNA endogenous controls. Quantification cycles of (**A**) miR-320/U48 in T-cells and **(B**) miR-942/U44 in whole blood cells of patients before (T1) and after (T2) elective cardiopulmonary bypass surgery. Data are shown as mean with range. n = 11/9 (T-cells/whole blood cells).

For further validation of the identified reference genes, we prospectively analysed two additional independent cohorts of patients undergoing elective cardiopulmonary bypass (CPB) surgery (patient characteristics are shown in Supplementary Tables 8 and 9). A pronounced immune dysfunction affecting both innate and adaptive immunity has been demonstrated for these patients immediately after surgical intervention^31–35^. As we have recently published, both septic patients and patients after CPB display comparable T-cell phenotypes with upregulation of immunosuppressive markers, rendering these cohorts ideal for validation of the identified reference genes^16,31^. As depicted in Fig. 7, expression of miR-320/U48 in T-cells and miR-942/U44 in whole blood cells remained stable before (T1) and after (T2) CPB, thus confirming their suitability to serve as endogenous controls.

### Impact of the choice of reference genes on miRNA quantification

**Figure 8:**
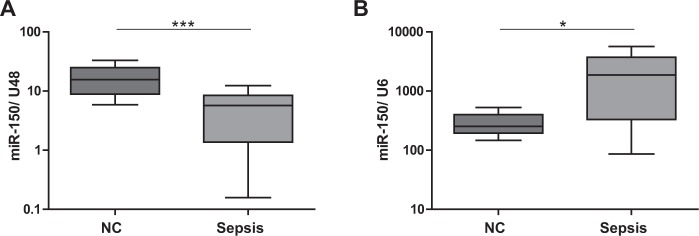
Effects of internal controls on microRNA quantification in sepsis. MiRNA-150 expression in primary human T-cells of septic patients as compared to healthy controls. Expression level of miR-150 T-cells of septic patients and healthy controls was measured by TaqMan miRNA assays relative to (**A**) U48 and (**B**) U6. Data are shown as median, 25^th^ and 75^th^ percentile and outliers, n = 5/16 (NC/Sepsis), performed in duplicates. *p < 0.05, ***p < 0.001. Quantification cycle (Cq) values were in the range of 21 (NC) and 23 (Sepsis) for miR-150, 30 (NC) and 33 (Sepsis) for U6 and 25 (NC/Sepsis) for U48, respectively.

To evaluate the effects of different reference genes on miRNA expression analysis, we quantified immuno-miR-150 in sepsis using best-performing U48 and worst ranked U6 as exemplary housekeeping genes. Consistent with recently published data^16^, miR-150 was significantly downregulated in septic patients as compared to healthy controls in case of using U48 for normalization (Fig. 8A). Remarkably, using snRNA U6 resulted in the opposite: a significant upregulation of miR-150 expression in sepsis was found (Fig. 8B). These divergent results highlight the crucial importance of using validated internal controls for qPCR-based miRNA expression analysis.

## Discussion

Sepsis is a clinical syndrome characterized by complex immune dysfunction with states of hyperinflammation, largely driven by innate immune cells, and immunosuppression, mainly affecting adaptive immunity^36^. Recent studies have pointed out that T-cell driven immunoparalysis is the leading cause of death in sepsis^29^. While the commonly used biomarkers, e.g. C-reactive Protein, Interleukin-6, and Procalcitonin, are able to detect hyperinflammation, effective tools for early detection and consecutive monitoring of immunosuppression are lacking^37^. At this point, miRNAs are being discussed to fill the gap^16^. MiRNAs have emerged as essential regulators of human immune responses. Moreover, they exhibit essential features -like cell-type and disease-specific expression and high stability- to render them ideal biomarkers^12,15^. Even though miRNAs have evolved as promising diagnostic tools in sepsis in recent years, their implementation into clinical routine is still pending^7^. This is -at least partially- due to the lack of valid reference genes, which impedes reliable miRNA quantification in sepsis.

To date, qPCR is the method of choice for quantification of miRNA expression, enabling a fast, accurate and reliable detection^25,38^. Suitable normalization strategies, however, are a pivotal prerequisite to generate valid results. To adjust for even slight sampling differences in RNA quantity and quality or reverse transcription efficiency, normalization to stably expressed reference genes is indispensable to actually identify variations in gene expression due to experimental conditions^24,39–42^. For miRNA quantification, small RNAs such as small nuclear RNA (<350 nt, e.g. U6) and small nucleolar RNA (~60–200 nt, e.g. U44/SNORD44, U47/SNORD47, U48/SNORD48), or other miRNAs are mainly used as endogenous controls^17–23,43–50^. We here validated endogenous controls for miRNA quantification in sepsis to provide reliable internal controls for current and future sepsis studies, which -in the majority- use healthy subjects as controls (Supplementary References 3–20). To this end, four widely used and commonly “accepted” yet not validated reference genes (U6, U44, U47, U48) as well as three potentially “new” miRNAs (miR-30c-1*, miR-320a, miR-942) were validated in T-cell and in whole blood cell samples both of healthy subjects and of septic patients. MiRNAs were identified via array analysis and have not been assigned to any regulatory role in immune cells, thus implying stable expression.

For T-cell miRNA quantification, miR-320 and U48 revealed as the two best reference genes, performing equally well. The most suited endogenous controls for whole blood cell miRNA quantification were miR-942 and U44, displaying stability values in close proximity. For these candidate genes, high consensus was obtained in all applied algorithms. The next placed candidate genes showed markedly inferior results. Importantly, the frequently used reference gene U6 was identified as the least stable expressed candidate gene in all cells and conditions tested, clearly showing that its use for normalization in sepsis is inadequate^20,51,52^. In order to further clarify this crucial point, we quantified immuno-miR-150 and could display that completely misleading results were obtained when U6 was applied for normalization.

Notably, our study could not find “universal” endogenous controls suitable for both T-cells and whole blood cells in sepsis. This is not surprising as whole blood samples are composed of a variety of different cell types exhibiting substantial differences in their transcriptome and thus miRNAome profiles. Our findings emphasize the necessity to use distinct miRNA internal reference genes for miRNA quantification in T-cell and/or whole blood samples in sepsis.

Several algorithms for analysis of qPCR results and identification of suitable endogenous controls are currently available, based on various principles and using distinct equations^25^. As a consequence, results may differ considerably depending on the respective algorithm^53^. Additionally, BestKeeper software is not specifically designed to construct a hierarchy of genes, although SD values and coefficient of variation are commonly used to rank reference candidates. Therefore, the use of multiple validation principles should be attempted^42,54^. Strikingly, we obtained similar results in all software algorithms used, which additionally supports our findings. Remarkably, the two best internal controls for T-cells (miR-320/U48) and whole blood cells (miR-942/U44) exhibit nearly 100% consensus amongst all normalization programs (i.e. being ranked first or second). Further prospective validation of the here-identified reference genes in independent cohorts of patients undergoing CPB surgery confirmed expression stability of miR-320/U48 and miR-942/U44 in T-cells and whole blood cells, respectively, in a highly controlled setting of immune dysfunction.

Regarding the optimal number of reference genes, pairwise variation calculations and accumulated standard deviations significantly improved in both sample groups when a second internal control was applied. Use of more than two reference genes had - if any - only minor benefits. The use of only one endogenous control may be acceptable if its use is restricted to the here-analysed experimental environment. Two reference genes may significantly reduce errors and should generally be recommended^25,27^. For individual studies it may be necessary to reasonably compromise on adequate normalization, sample availability, economical issues and technical execution.

Several studies have been performed to identify suitable normalizers for quantification of -mostly circulating- miRNA in different diseases. Using multiple algorithms and recommending at least two housekeepers is a common ground for most of these investigations^50,52,55–57^. To date, only one study has evaluated reference genes in plasma in a very small number of patients with septic shock^58^. However, both the small sample size and a potential distortion of results by miRNAs induced or released by co-morbidities may hamper the validity of this approach.

Thus, to the best of our knowledge, we here provide for the first time reliable identification of suitable controls for miRNA quantification in immune cells in sepsis. All patient samples have been acquired at the onset of sepsis. As the validated miRNAs remain stable even in the initial activation of immune cells -which profoundly alters T-cell transcriptional activity- it can be assumed that these internal controls also remain stable in the course of sepsis^4^.

Taken together, we recommend the use of miR-320 and U48 in T-cells, and miR-942 and U44 in whole blood cells as suitable endogenous controls for miRNA quantification in sepsis. These combinations feature important advantages: (i) they displayed outstanding stability values, (ii) they both combine different classes of RNAs - miRNA and snoRNA - thus ameliorating potential biases due to biochemical differences, and (iii) they do not comprise of miRNAs of the same miRNA family or miRNA cluster, avoiding similar regulation mechanisms in the context of experimental conditions.

Consensus of applied housekeepers for normalization might enable reliable research on miRNAs in sepsis, especially with respect to their use as biomarkers. In this regard, miRNAs might even prove suitable for point-of-care applications and thus could improve current treatment strategies in the future.

## Methods

### Blood sampling

Blood samples from septic patients were withdrawn immediately after diagnosis of sepsis or septic shock (according to the Sepsis-2 criteria^59^) and admission to the intensive care unit, before antibiotic or steroid treatment was initiated. Retrospective analysis also confirmed that all patients were meeting the Sepsis-3 definitions^1,60^. Patient analysis has been performed between 2011 and 2017, healthy age-adjusted controls (similar mean age of the compared groups) were sampled in 2011 and 2018. Two independent patient and age-adjusted control groups have been evaluated for primary T-cell and whole blood cell analysis, respectively. Previous antibiotic or steroid treatment, age younger than 18 years, history of malignant diseases, pre-existing immunodeficiency or autoimmune diseases have been defined as patient exclusion criteria. Research has been performed in accordance to the Declaration of Helsinki (ethical principles for medical research involving human subjects). Informed consent was obtained from all patients and healthy volunteers. The protocol for this prospective study was approved by the Institutional Ethics Committees of the Ludwig-Maximilian-University Munich, Germany (No. 107-11 and No. 287-13; approved in 2006 and in 2013, respectively), of the University Hospital of Jena (No. 2007-004333-42, local amendment for Munich University Hospital 2242-03/08), and by the Ethics Committee of ATTIKON University Hospital (approval 5/2008). The study protocol for the independent validation cohorts was also approved by the Institutional Ethics Committees of the Ludwig-Maximilians-University Munich (No. 17–241).

### T-cell isolation, culture and stimulation

Peripheral blood mononuclear cells (PBMCs) were obtained by density centrifugation (Histopaque 1077, Sigma-Aldrich, St. Louis, MO, USA). T-cell isolation has been performed by untouched negative magnetic cell separation (Pan T Cell Isolation Kit, Cat. # 130-096-535, Miltenyi Biotec, Bergisch Gladbach, Germany), using an AutoMACS Pro Separator (Cat. # 130-092-545, Miltenyi Biotec, Bergisch-Gladbach, Germany) according to the manufacturer’s instructions. A ViCell analyzer (Beckman Coulter, Fullerton, CA, USA) was utilized to assess cell number and viability. Only samples exceeding a cell viability of 90% were included into the analysis. T-cells from healthy donors were cultured in RPMI-1640 (Sigma-Aldrich, St-Louis, MO, USA) supplemented with 10% heat-inactivated fetal calf serum (Biochrom, Berlin, Germany), 1% HEPES (Sigma-Aldrich, St. Louis, MO) and 1% L-glutamine (Life Technologies, Carlsbad, CA, USA) for 24 hours. For T-cell stimulation, cells have been incubated using anti-CD3/CD28 Dynabeads (Thermo Fisher Scientific, Waltham, MA, USA) at a bead-to-cell ratio of 1:1 for 24 hours. Figure S2 displays efficiency of T-cell stimulation and provides data to exclude non-specific activation of naive T-cells.

### Whole blood analysis

For whole blood analysis, blood samples were collected using the PAXgene Blood RNA Tube (PreAnalytiX, Hombrechtikon, Switzerland) as to the manufacturer’s instructions.

### RNA-Isolation

Total RNA was isolated from primary T-cells using the mirVana miRNA Isolation Kit (Thermo Fisher Scientific, Waltham, MA, USA) with subsequent DNase treatment (Turbo DNase, Thermo Fisher Scientific, Waltham, MA, USA). Total RNA from whole blood samples was purified using the PAXgene Blood RNA Kit (PreAnalytiX, Hombrechtikon, Switzerland). All isolation procedures have been performed according to the respective manufacturer’s instructions. RNA quantity and purity were measured using a NanoDrop 2000 spectrophotometer (Thermo Fisher Scientific, Waltham, MA, USA). Only samples exhibiting A260/A280 ratios between 1.70 and 2.10 have been further analysed. RNA storage has been performed at −80 °C. No difference in RNA quality of older samples has been detected over time. For additional validation of sample integrity, Fig. S3 depicts U47 quantification cycles of samples independently analysed in 2011, 2013, 2017 and 2018.

### miRNA Microarray

Total (T-cell derived) RNA from seven patients and from seven controls was used for miRNA Microarray analysis (miRCURY LNA™ microRNA Array, Exiqon A/S, Vedbaek, Denmark), as to the manufacturer’s recommendations.

### miRNA quantification

Four commonly applied miRNA housekeeping genes and three newly identified miRNAs were selected as candidate reference genes (Table 1). Expression of miRNAs was quantified using a LightCycler 480 instrument (Roche Diagnostics, Mannheim, Germany). In all reactions, identical amounts of extracted total RNA (6 ng) were reversely transcribed using TaqMan miRNA assays (Thermo Fisher Scientific, Waltham, MA, USA). Negative controls using only purified water were included to prevent any contamination. To account for inter-run and intra-run variations, all experiments have been performed in technical duplicates and additional calibration has been applied, respectively. The cycling conditions comprised initial denaturation a 95 °C for 10 min followed by 50 amplification cycles at 95 °C for 15 s, 60 °C for 60 s and 40 °C for 30 sec. Determination of raw quantification cycles (Cq) has been performed by the LightCycler software using the second derivative maximum method. A quantification cycle (Cq) cut-off has been defined (Cq 40) and all Cq values beyond this cut-off have been considered unspecific. For all TaqMan miRNA assays, amplification efficiencies have been determined by calculating calibration curves from 10-fold dilution series using the equation E = −1 + 10^(−1/slope)^ (See Table S1 for efficiency values and amplification factors for each TaqMan miRNA assay).

### Statistical analysis

Student’s *t*-test or Mann-Whitney U tests, as appropriate, served for comparisons. Normal distribution was tested using the Kolmogorov-Smirnov test. The quantified array signals were background corrected (Normexp with offset value 10 - Convolution model described by Ritchie *et al*.^61^) and normalized using the global Lowess (LOcally WEighted Scatterplot Smoothing) regression algorithm. The obtained values were further analyzed using two-sided Student’s t-test. Analysis of expression stability of potential reference genes was evaluated by Excel-based software tools BestKeeper^41^, NormFinder^40^ and geNorm^24^. NormFinder add-in calculates intergroup and intragroup variations by applying a model-based approach. Both variations are integrated into a gene stability value. BestKeeper algorithm displays expression stability based on Cq variations and pair-wise correlation analyses. Ranking of genes was performed based on SD values. In case of equal SD, coefficient variation served as additional ranking value. GeNorm gene expression stability is indicated by the average pairwise variation of each reference gene with all other candidate genes and additional ranking of genes by repeated stepwise exclusion of the worst performing gene and recalculation of stability values. For NormFinder and geNorm analysis, raw Cq data has been transformed into relative quantities by the comparative ∆CT method^62^. To calculate accumulated standard deviations of each candidate gene GenEx 6.1 (MultiD Analyses AB, Gothenburg, Sweden) was used according to the developer’s instructions. Statistical analysis of qPCR results was performed using Microsoft Excel 365 (Microsoft Corporation, Redmond, WA, USA) and GraphPad Prism 5.01 (GraphPad Software, Inc., USA). Vector artwork has been designed using Adobe Illustrator CS5.1 (Adobe Systems Inc., San Jose, CA, USA). If not stated otherwise, no missing values are reported.

## Supplementary information

Supplementary Information

## Data Availability

The complete data generated and analysed for this study is implemented into this publication and the Supplementary Files.
