# Supplementary material for: Identification of suitable controls for miRNA quantification in T-cells and whole blood cells in sepsis
Source: Sci Rep. 2019 Oct 31;9:15735. doi: 10.1038/s41598-019-51782-w (PMC6823537; doi:10.1038/s41598-019-51782-w)
Supplement: Supplementary file 1 — Supplementary Information [file 41598_2019_51782_MOESM1_ESM.pdf]

Supplementary

Identification of suitable controls for miRNA quantification in T-cells and whole blood cells in sepsis

Simon Hirschberger[1,2], Max Hübner[1,2], Gabriele Strauß[1,2], David Effinger[1,2], Michael Bauer[3,4], Sebastian Weis[3,4,5], Evangelos J. Giamarellos-Bourboulis[6], Simone Kreth[1,2, *]

[1] Department of Anaesthesiology and Intensive Care Medicine, University Hospital, Ludwig Maximilian University (LMU), Munich, Germany
[2] Walter-Brendel-Center of Experimental Medicine, Ludwig Maximilian University (LMU), Munich, Germany
[3] Department of Anaesthesiology and Intensive Care Medicine, Friedrich-Schiller University, Jena, Germany
[4] Center for Sepsis Control and Care, Jena University Hospital, Jena, Germany.
[5] Institute for Infectious Disease and Infection Control, Jena University Hospital, Jena, Germany
[6] 4th Department of Internal Medicine, ATTIKON University Hospital, National and Kapodistrian University of Athens, Athens, Greece.

*** Corresponding author:**
Simone Kreth, M.D., Ph.D.
Department of Anaesthesiology and Intensive Care Medicine, University Hospital, Ludwig Maximilian University (LMU), Marchioninistraße 15, 81377 Munich, Germany
Email: simone.kreth@med.uni-muenchen.de,
Phone: +49 89 4400 76423
Fax: +49 89 4400 78886

Supplementary Figures

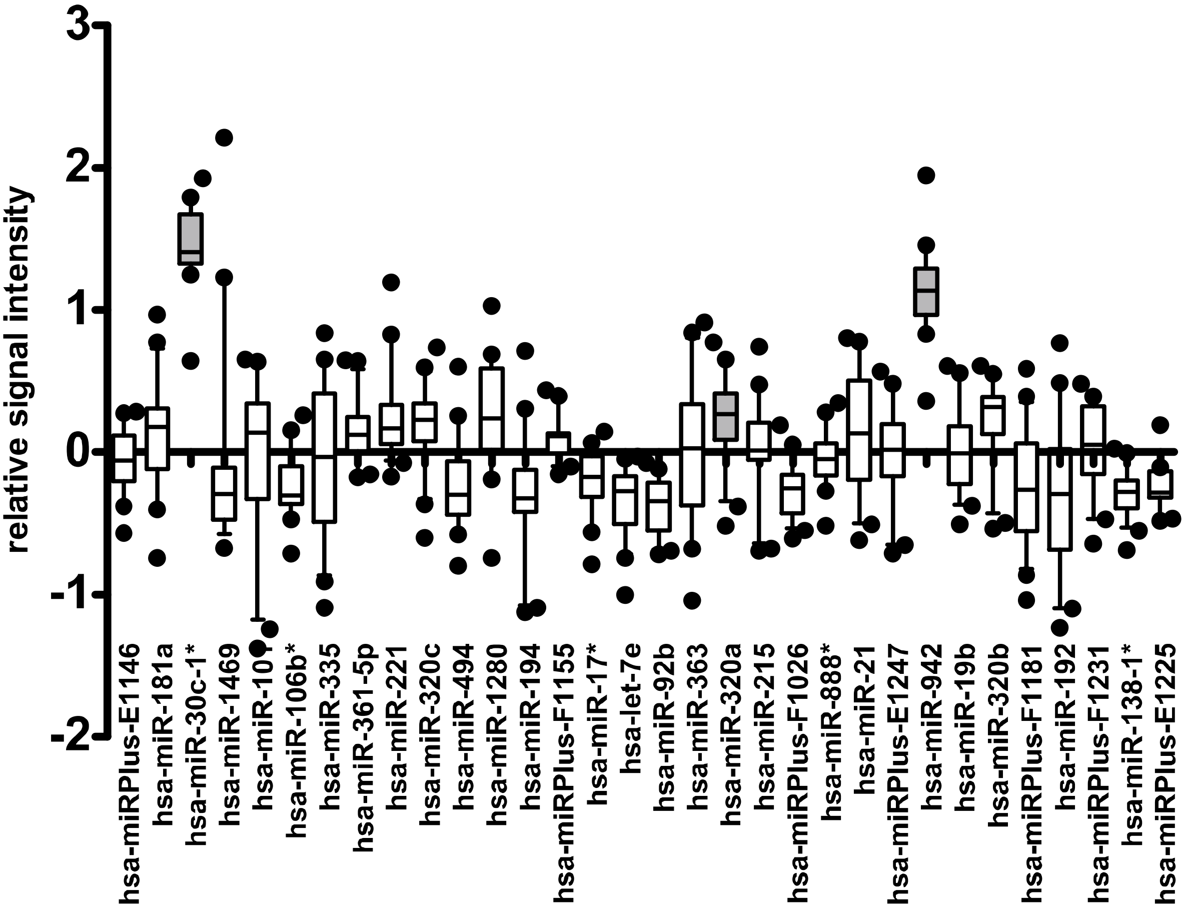

Figure S1 Potential miRNA reference genes: MicroRNA microarray analysis of RNA isolated from T-cells of septic patients and native (unstimulated) and CD3/28-activated T-cells from healthy volunteers, *n=7/7/7* (Sepsis/ NC/ NC stimulated). MicroRNAs not differentially regulated (p > 0.05) and detectable in at least 19/21 samples are depicted according to their relative signal intensity. Selected candidate miRNAs are highlighted.

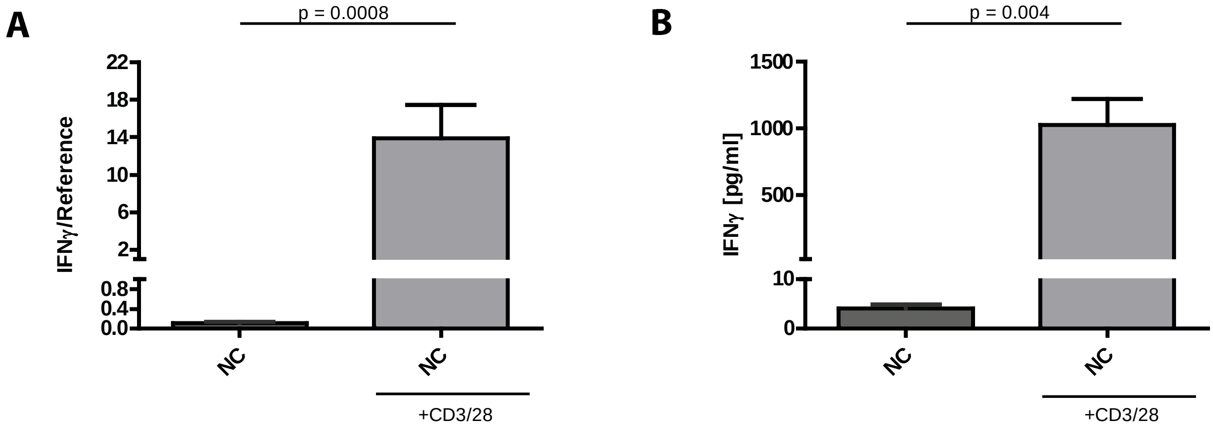

Figure S2 Efficiency of T-cell stimulation: Interferon γ (IFN-γ) expression in stimulated and native T-cells of healthy controls. (A) IFN-γ mRNA and (B) IFN-γ protein levels in +/- CD3/28-activated Pan T-cells of healthy volunteers were measured by qPCR (relative to reference genes SDHA and TBP) and IFN-γ enzyme-linked immunosorbent assay (ELISA), respectively. Data are presented as mean with range/ mean with standard error mean, measurements were performed in duplicates/ triplicates (qPCR/ ELISA). Quantification cycle (Cq) values for IFN-γ were in the range of 31 (NC) and 24 (NC + CD3/28). n = 14/ 6 (qPCR/ ELISA).

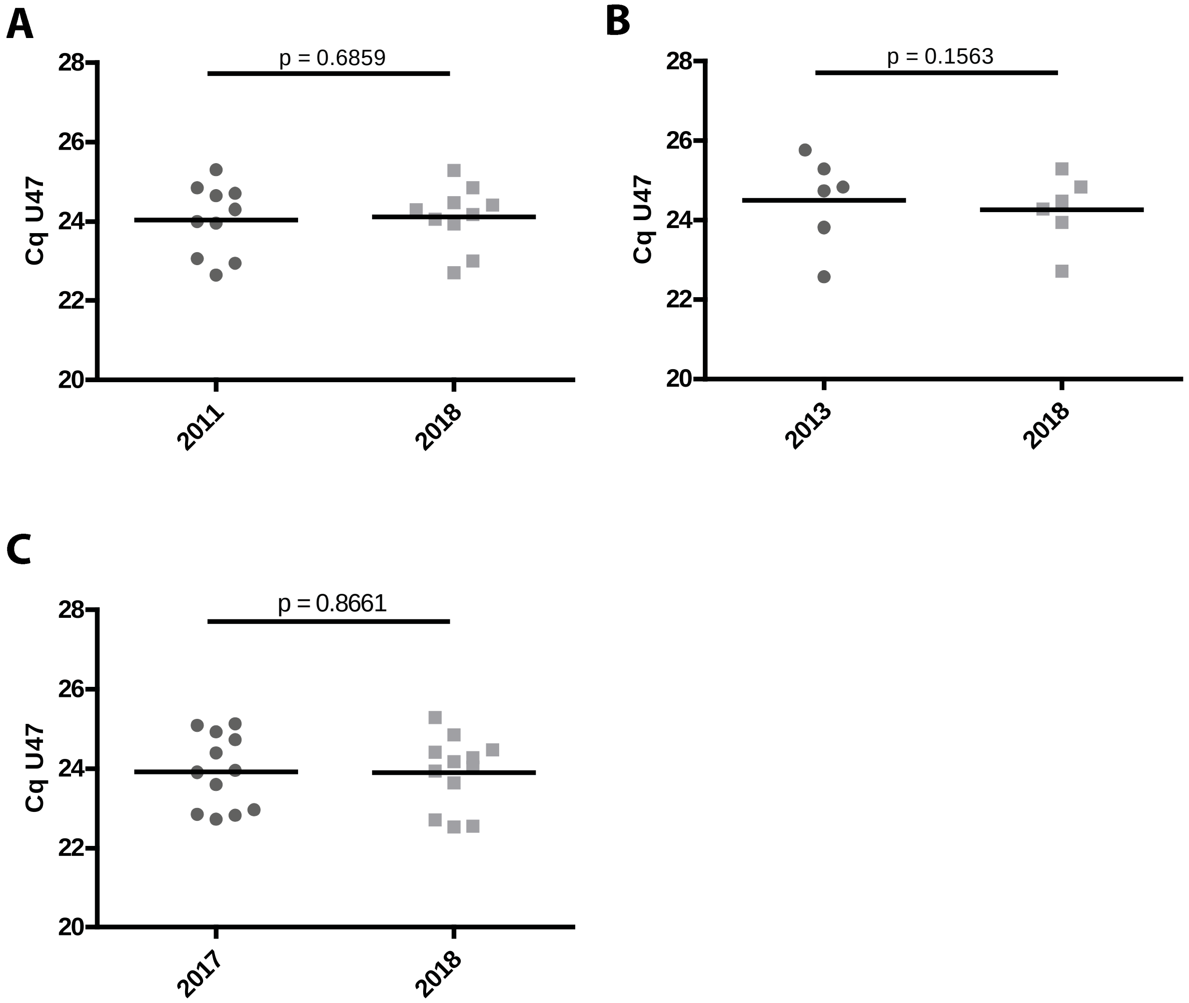

Figure S3 RNA sample integrity over time: Raw quantification cycles of U47 in septic patients were measured by TaqMan miRNA assay in (A) 2011, (B) 2013 and (C) 2017, compared to measurements in 2018. The same RNA samples have been used for transcription and RT-qPCR in the years 2011, 2013, 2017 and 2018, respectively. All experiments performed in duplicates, n = 10 /6 /12 (2011/ 2013/ 2017).

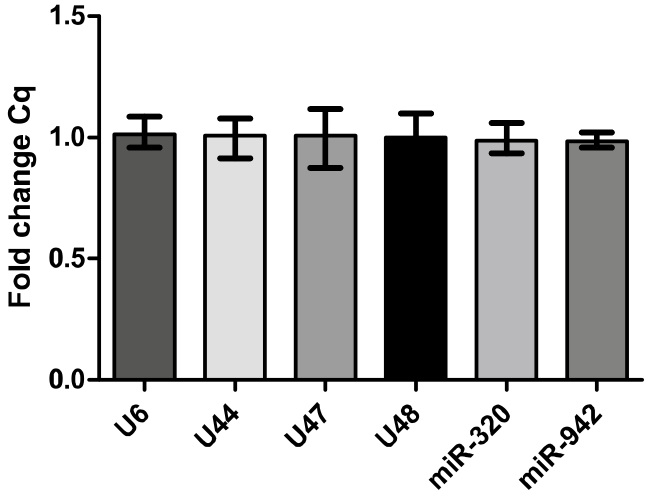

Figure S4 MicroRNA fold change of stimulated primary human T-cells: MicroRNA fold change expression of CD3/CD28-activated primary T-cells of healthy donors compared to native T-cells of the same healthy controls. n = 15.

Supplementary Tables

Table S1 Amplification efficiencies

TaqMan Assay	Slope	Intercept	E	R^2	Amplification factor
RNU6B	3.0105	31.451	114.87	0.9817	2.15
RNU44	2.7209	27.721	133.09	0.9749	2.33
U47	2.9075	26.523	120.77	0.9899	2.21
RNU48	3.1510	24.632	107.66	0.9995	2.08
hsa-miR-320a-3p	3.1098	21.773	109.68	0.9990	2.10
hsa-miR-942-5p	3.3368	26.284	99.38	0.9992	1.99

Table S2 Patient Characteristics: primary T-cell samples

**n**	18
**Gender (male/female)**	11/7
**Age, years (mean ± SD)**	49.2 (± 11.9)
**Septic shock**	7 (38.9%)
**Sequential organ failure score (mean ± SD)**	10.6 (± 5.5)
**Nonsurvivors**	5 (27.8%)

Table S3 Characteristics of healthy controls: primary T-cell samples

**n**	17
**Gender (male/female)**	9/8
**Age, years (mean ± SD)**	43.5 (± 14.0)

All healthy volunteers were nonsmokers, without suspect of any acute or chronic disease, blood count and electrolytes within normal range.

Table S4 Patient Characteristics: whole blood cell samples

**n**	17
**Gender (male/female)**	11/6
**Age, years (mean ± SD)**	68.1 (± 13.7)
**APACHE II score (mean ± SD)**	22.0 (± 10.0)
**Nonsurvivors**	9 (52.9%)

Table S5 Characteristics of healthy controls: whole blood cell samples

**n**	15
**Gender (male/female)**	9/6
**Age, years (mean ± SD)**	64.5 (± 21.3)

All healthy volunteers were nonsmokers, without suspect of any acute or chronic disease, blood count and electrolytes were within normal range.

Table S6 Cq fold change of stimulated healthy controls

**U6**	**U44**	**U47**	**U48**	**miR-320**	**miR-942**
0,97835069	0,93533588	0,91173518	0,9340602	0,97483382	0,99311727
0,97667343	1,04488217	1,05907818	1,00766866	0,98383354	1,01905748
1,04772889	0,91466787	0,87457975	0,9513057	0,99767848	0,99527603
0,97851948	0,98437995	0,96028972	1,01041679	0,96011391	0,95851542
0,96666425	0,96583102	0,96380033	0,95395977	0,93386547	0,96060154
1,01482987	1,05631208	1,00558728	0,98656954	0,98018866	0,97185569
0,95644359	0,94946411	0,97773036	0,96520895	0,96195933	0,96110881
1,03051313	1,01756078	1,0202875	1,00961606	0,99959155	0,97474071
1,038721	1,03583244	1,06439916	1,04340812	1,02444032	1,00386318
1,05371945	1,0746986	1,06260132	1,03711895	1,00690906	0,99644451
1,04867998	1,04749733	1,03826569	1,0298375	0,99062295	0,9650736
1,00552393	1,00362118	1,01753015	0,9905216	0,98055865	0,98558751
0,99323547	0,98538637	0,99886412	1,00501194	0,9596919	0,98182042
1,08671106	1,07811042	1,11834852	1,09812129	1,05892657	1,00515225
1,01053688	1,03454148	1,04152277	0,99567224	0,99029385	0,99375702

Fold Change of raw quantification cycles of CD3/28 stimulated T-cells compared to native T-cells of the same healthy controls.

Table S7 qRT-PCR Primers and Probes for mRNA quantification

Target/ Probe/ Primer direction	Primer sequence
SDHA #132 FW	5'-GAG GCA GGG TTT AAT ACA GCA- 3'
SDHA #132 RV	5'-CCA GTT GTC CTC CTC CAT GT- 3'
TBP #87 FW	5'-GAA CAT CAT GGA TCA GAA CAA CA- 3'
TBP #87 RV	5'-ATA GGG ATT CCG GGA GTC AT- 3'
IFN-γ #21 FW	5' -GGC ATT TTG AAG AAT TGG AAA G- 3'
IFN-γ #21 RV	5' -TTT GGA TGC TCT GGT CAT CTT- 3'

Table S8 Patient Characteristics: independent validation cohort T-cells | cardiopulmonary bypass surgery.

Characteristics	n
**Age, years (mean ± SD)**	63.3 (± 11.1)
**Gender (male/ female)**	6/5
**CPB-time, min (mean ± SD)**	135.9 (± 41.3)
**Total time of surgery, min (mean ± SD)**	297 (± 154.3)
**Type of cardiac surgery**	n
**Coronary artery bypass grafting**	3
**Heart valve surgery**	6
**Combined coronary artery bypass grafting and heart valve surgery**	2

Table S9 Patient Characteristics: independent validation cohort whole blood cells | cardiopulmonary bypass surgery.

**Characteristics**	**n**
**Age, years (mean ± SD)**	69.6 (± 6.5)
**Gender (male/ female)**	6/3
**CPB-time, min (mean ± SD)**	97.1 (± 33.1)
**Total time of surgery, min (mean ± SD)**	212.3 (± 42.5)
**Type of cardiac surgery**	n
**Coronary artery bypass grafting**	7
**Heart valve surgery**	2
**Combined coronary artery bypass grafting and heart valve surgery**	0

Supplementary Methods:

mRNA quantification

Expression of Interferon γ (IFN-γ) mRNA was determined using a LightCycler 480 instrument (Roche Diagnostics, Mannheim, Germany) as previously described [1]. Succinate dehydrogenase subunit A (SDHA) and TATA Box Binding Protein (TBP) were used as reference genes in all experiments [2]. In all reactions, equal amounts of RNA were used (for mRNA transcription 1000 ng of total RNA). Mean Target/Reference and Target/Reference standard deviation has been calculated. Determination of quantification cycles has been performed by the LightCycler software using the second derivative maximum method. Quantification cycle (Cq) cut-offs have been defined for mRNA (Cq 35) quantification. Cq values beyond cut-offs have been considered unspecific. qRT-PCR Primers and Probes are depicted in Table S7.

Enzyme-Linked Immunosorbent Assay (ELISA)

T-cells were harvested after 24 hours of cultivation. Supernatant was immediately stored at - 80°C. For quantification of IFN-γ, 100 µL of cell culture supernatant were analyzed using a 96-well enzyme-linked immunosorbent assay (LEGEND MAX Human Interferon γ [IFNγ] ELISA Kit, Cat. No. 430107; BioLegend, San Diego, CA) according to the manufacturer's instructions.

Patient recruitment: validation cohort

Patients undergoing elective cardiopulmonary bypass (CPB) surgery have been enrolled (Patient characteristics are depicted in supplementary tables S8 and S9). Age <18 years, non-elective surgery, pregnancy, pre-existing malignancies, acute inflammation before surgery, organ transplantation and immunosuppression were defined as patient exclusion criteria. Prior to study enrolment, informed written consent was obtained from every patient. Blood was withdrawn before (T1) and after (T2) CPB.

Supplementary References:

1. Hübner, M. *et al.* Intronic miR-744 Inhibits Glioblastoma Migration by Functionally Antagonizing Its Host Gene MAP2K4. *Cancers* **10**, (2018).
2. Ledderose, C., Heyn, J., Limbeck, E. & Kreth, S. Selection of reliable reference genes for quantitative real-time PCR in human T cells and neutrophils. *BMC Res. Notes* **4**, 427 (2011).
3. Vasilescu, C. *et al.* MicroRNA fingerprints identify miR-150 as a plasma prognostic marker in patients with sepsis. *PLoS One* **4**, e7405 (2009).
4. Möhnle, P. *et al.* MicroRNAs 143 and 150 in whole blood enable detection of T-cell immunoparalysis in sepsis. *Mol. Med.* **24**, 54 (2018).
5. Ma, Y. *et al.* Genome-Wide Sequencing of Cellular microRNAs Identifies a Combinatorial Expression Signature Diagnostic of Sepsis. *PLoS ONE* **8**, e75918 (2013).
6. How, C.-K. *et al.* Expression profile of MicroRNAs in gram-negative bacterial sepsis. *Shock* **43**, 121–127 (2015).
7. Li, Y. *et al.* Plasticity of leukocytic exudates in resolving acute inflammation is regulated by MicroRNA and proresolving mediators. *Immunity* **39**, 885–898 (2013).
8. Tudor, S. *et al.* Cellular and Kaposi's sarcoma-associated herpes virus microRNAs in sepsis and surgical trauma. *Cell Death Dis.* **5**, e1559 (2014).
9. Roderburg, C. *et al.* Circulating microRNA-150 serum levels predict survival in patients with critical illness and sepsis. *PLoS One* **8**, e54612 (2013).
10. Tacke, F. *et al.* Levels of circulating miR-133a are elevated in sepsis and predict mortality in critically ill patients. *Crit. Care Med.* **42**, 1096–1104 (2014).
11. Wang, J.-F. *et al.* Serum miR-146a and miR-223 as potential new biomarkers for sepsis. *Biochemical and Biophysical Research Communications* **394**, 184–188 (2010).
12. Wang, H. *et al.* Evidence for serum miR-15a and miR-16 levels as biomarkers that distinguish sepsis from systemic inflammatory response syndrome in human subjects. *Clin. Chem. Lab. Med.* **50**, 1423–1428 (2012).
13. Wang, H.-J. *et al.* Four serum microRNAs identified as diagnostic biomarkers of sepsis. *J. Trauma Acute Care Surg.* **73**, 850–854 (2012).
14. Roderburg, C. *et al.* Elevated miR-122 serum levels are an independent marker of liver injury in inflammatory diseases. *Liver Int.* **35**, 1172–1184 (2015).
15. Wang, H., Yu, B., Deng, J., Jin, Y. & Xie, L. Serum miR-122 correlates with short-term mortality in sepsis patients. *Crit. Care* **18**, 704 (2014).
16. Wu Y. *et al.* [Relationship between expression of microRNA and inflammatory cytokines plasma level in pediatric patients with sepsis]. *Zhonghua Er Ke Za Zhi* **52**, 28–33 (2014).
17. Benz, F. *et al.* Circulating MicroRNA-223 Serum Levels Do Not Predict Sepsis or Survival in Patients with Critical Illness. *Disease Markers* **2015**, 1–10 (2015).
18. Puskarich, M. A. *et al.* Detection of microRNAs in patients with sepsis. *Journal of Acute Disease* **4**, 101–106 (2015).
19. Wang, H.-J. *et al.* Serum miR-122 levels are related to coagulation disorders in sepsis patients.

Clin. Chem. Lab. Med. **52**, 927–933 (2014).

20. Han, Y., Dai, Q.-C., Shen, H.-L. & Zhang, X.-W. Diagnostic value of elevated serum miRNA-143 levels in sepsis. *Journal of International Medical Research* **44**, 875–881 (2016).
